# Duration of COVID-19: Data from an Italian Cohort and Potential Role for Steroids

**DOI:** 10.3390/microorganisms8091327

**Published:** 2020-08-31

**Authors:** Damiano D’Ardes, Michela Pontolillo, Lucia Esposito, Mara Masciarelli, Andrea Boccatonda, Ilaria Rossi, Marco Bucci, Maria Teresa Guagnano, Claudio Ucciferri, Francesca Santilli, Marta Di Nicola, Katia Falasca, Jacopo Vecchiet, Thomas Schael, Francesco Cipollone

**Affiliations:** 1 “Clinica Medica” Institute, Department of “Medicine and Science of Aging”, “G. d′Annunzio” University, 66100 Chieti, Italy; espositolucia92@gmail.com (L.E.); andrea.boccatonda@gmail.com (A.B.); rossilaria91@gmail.com (I.R.); mbucci@unich.it (M.B.); guagnano@unich.it (M.T.G.); fra.santilli22@gmail.com (F.S.); francesco.cipollone@unich.it (F.C.); 2 Azienda Sanitaria Locale no. 2 Abruzzo Lanciano-Vasto-Chieti, 66100 Chieti, Italy; michela.pontolillo@gmail.com (M.P.); claudio.ucciferri@unimol.it (C.U.); katia.falasca@unich.it (K.F.); jacopo.vecchiet@unich.it (J.V.); direzione.generale@asl2abruzzo.it (T.S.); 3 Unit of Infectious Diseases, Department of “Medicine and Science of Aging”, “G. d′Annunzio” University, 66100 Chieti, Italy; 4 Unit of Hygiene and Preventive Medicine, Department of “Medicine and Science of Aging”, “G. d′Annunzio” University, 66100 Chieti, Italy; masciarellimara@gmail.com; 5 Laboratory of Biostatistics, Department of “Medical, Oral and Biotechnological Sciences”, “G. d’Annunzio” University, 66100 Chieti, Italy; mdinicola@unich.it

**Keywords:** COVID-19, SARS-CoV-2, viral shedding, steroids

## Abstract

The diffusion of SARS-CoV-2, starting from China in December 2019, has led to a pandemic, reaching Italy in February 2020. Previous studies in Asia have shown that the median duration of SARS-CoV-2 viral shedding was approximately 12–20 days. We considered a cohort of patients recovered from COVID-19 showing that the median disease duration between onset and end of COVID-19 symptoms was 27.5 days (interquartile range (IQR): 17.0–33.2) and that the median duration between onset of symptoms and microbiological healing, defined by two consecutive negative nasopharyngeal swabs, was 38 days (IQR: 31.7–50.2). A longer duration of COVID-19 with delayed clinical healing (symptom-free) occurred in patients presenting at admission a lower PaO_2_/FiO_2_ ratio (*p* < 0.001), a more severe clinical presentation (*p* = 0.001) and a lower lymphocyte count (*p* = 0.035). Moreover, patients presenting at admission a lower PaO_2_/FiO_2_ ratio and more severe disease showed longer viral shedding (*p* = 0.031 and *p* = 0.032, respectively). In addition, patients treated with corticosteroids had delayed clinical healing (*p* = 0.013).

## 1. Introduction

The COVID-19 pandemic started late 2019 in China and has represented a substantial change in the health of populations worldwide.

Since December 2019, multiple cases with unexplainable pneumonia were reported in hospitals in Wuhan city. The cases had history of exposure to a large seafood market in Wuhan city, Hubei province, China. It has been confirmed as an acute respiratory infection caused by a novel coronavirus, named SARS-CoV-2. So far, this disease has rapidly spread to other areas, reaching Italy in February 2020.

Coronavirus belongs to Orthocoronavirinae of the family of Coronaviridae in the order Nidovirales, which are responsible for infections in the respiratory and gastrointestinal tracts. SARS-CoV-2 is a new enveloped beta-coronavirus that has a single-stranded positive-sense RNA genome [1].

The first genome sequence for SARS-CoV-2 was released on 10 January 2020 and allowed for rapid development of several sensitive and specific real-time reverse-transcriptase-polymerase-chain-reaction (RT-PCR) assays. A lot of laboratories worldwide are now able to test for SARS-CoV-2, and assays can detect a few copies of SARS-CoV-2 per reaction that do not show a cross-reaction with other human coronaviruses, even if specificity and sensitivity of the tests are not exactly known [2].

Although the outbreak is likely to have started from a zoonotic transmission event associated with a large seafood market that also traded in live wild animals, it soon became clear that efficient person-to-person transmission was also occurring [3].

Therefore, the main infection source was patients with pneumonia infected by SARS-CoV-2. Respiratory droplet transmission is the main route of transmission, and it can be also transmitted through aerial droplets and contact. In the absence of a safe and effective vaccine as well as a lack of specific drug treatment, the only solution is preventing its transmission; performing general education; and implementing appropriate prevention, control and treatment [4].

The most effective measure to prevent the spread of the infection is based on isolation of infected people and their close contacts. The very first observation of the incubation period of SARS-CoV-2 came from the National Health Commission of China, reporting an incubation time between 1 and 14 days [5].

SARS-CoV-2 RNA has been identified by RT-PCR in respiratory tract samples 1–2 days prior to symptom onset and can also be detected in whole blood, saliva, faeces and urine [6,7,8,9].

Viral RNA loads by RT-PCR seem to be higher in sputum than in throat swabs, suggesting that the type of sample may also influence the outcome of the test [10,11].

Patients with severe COVID-19 seem to have a significantly higher viral load and a longer period of viral shedding than mild cases [12].

Prolonged viral RNA shedding has been reported from throat swabs for up to 37 days among adult patients and in faeces for over 1 month after illness onset in children [13].

Moreover, several studies in Asia have shown that median duration of SARS-CoV-2 viral shedding was approximately 12–20 days [7,14], and prevention measures to limit viral spread were generally established referring to this timing. However, the duration of viral shedding in adults with COVID-19 outside Asia has not been well explored.

## 2. Materials and Methods

### 2.1. Study Design and Population

This retrospective, single-centre cohort study included patients with COVID-19 hospitalized in the Internal Medicine and Infectious Disease Units of the Chieti University Hospital in Abruzzo region, Southern Italy, from 2 March 2020 to 4 April 2020. The study was performed in accordance with the principles embodied in the Declaration of Helsinki, and all participants provided written informed consent.

All adult patients were diagnosed with COVID-19 according to World Health Organization (WHO) interim guidance: they had clinical symptoms of COVID-19 and confirmation of SARS-CoV-2 infection through instrumental signs and a positive result on RT-PCR assays of nasopharyngeal swab specimens.

The specific inclusive criteria were as follows: (1) patients confirmed by positive detection of SARS-CoV-2 RNA from nasopharyngeal/throat swabs by RT-PCR with clinical data suggesting for COVID-19, (2) patients aged more than 18 years old and (3) patients with a known date of performing different RT-PCR assays.

We followed the patients up to clinical and microbiological healing. Clinical healing was defined by apyrexia, no need for oxygen therapy, normalization of inflammation indices and subjective well-being. Microbiological healing was certified by two consecutive negative nasopharyngeal swabs, performed at least 48 h apart.

Nasopharyngeal specimens were obtained for SARS-CoV-2 RT-PCR reexamination approximately every three days day after clinical remission of symptoms, including fever, cough and dyspnoea.

Epidemiological, demographic, clinical, laboratory, treatment and outcome data were extracted from medical records using a standardized data collection form. All data were checked by two physicians (M.P. and L.E.), and a third researcher (D.D.) adjudicated any difference in interpretation between the two primary investigators.

### 2.2. Statistical Analysis

Quantitative variables were summarized as median and interquartile range (IQR), while quantitative data were reported as frequency and percentage. Departures from normal distribution were evaluated for each variable using Shapiro–Wilk’s test.

Linear mixed models were fitted to test the effect of patient’s characteristics (as independent variables) on time of clinical and microbiological healing.

All tests were two-sided, and a level of statistical significance was set at *p* < 0.05. All the statistical analyses were performed using R software environment for statistical computing and graphics version 3.5.2 (R Foundation for Statistical Computing, Vienna, Austria. https://www.R-project.org/).

## 3. Results

### Demographic and Clinical Characteristics of Patients

A total of 88 patients with laboratory-confirmed COVID-19 were screened. We excluded 15 patients who died from complications of COVID-19 during hospitalization. The characteristics of the remaining 73 patients are listed in Table 1.

The median age in years was 63 (IQR: 52.0–76.0), with 43 men (58.9%) and 30 women (41.1%). Hypertension was present in 22 patients (30.1%), 4 patients had diabetes (5.5%) and 4 patients had coronary heart disease (5.5%). Chronic obstructive pulmonary disease was present in 2 patients (2.7%). Seven patients (9.6%) showed atrial fibrillation, and 6 patients had chronic kidney disease (8.2%). Moreover, 7 patients were obese (9.6%).

Concerning COVID-19 severity, only one patient was asymptomatic (1.4%), 2 patients had mild symptoms (2.7%), the most patients showed moderate pneumonia (33 patients; 45.2%) and severe pneumonia (34 patients; 46.6%), and only 3 patients developed acute respiratory distress syndrome (ARDS; 4.1%).

All patients received hydroxychloroquine (200 mg twice daily) and lopinavir–ritonavir (400 mg twice daily of lopinavir and 100 mg twice daily of ritonavir). The patients provided written informed consent for the off-label use of the drugs.

In addition, some patients were also treated with anti-cytokine or Janus kinase inhibitors drugs. Twenty-two patients (25%) were treated with tocilizumab, a monoclonal antibody that inhibits interleukin 6, administered with intravenous injection in two doses of 8 mg/kg each; 20 patients (22.72%) were treated with canakinumab, a human monoclonal antibody against IL-1β, administered subcutaneously in a single 300 mg dose; and 18 patients (24.7%) were treated with intravenous administration of corticosteroids in addition to other supportive therapies.

The median ratio of arterial oxygen partial pressure to fractional inspired oxygen (PaO_2_/FiO_2_) at admission was 242.0 (IQR: 186.5–304.5). The patients were admitted with a median value of lymphocytes of 990.0 cells/uL (IQR: 682.5–1260.0) and showed 1320.0 cells/uL (IQR: 1000.0–1680.0) at day 10. Median C-reactive protein was 65.0 mg/L (IQR: 30.8–148.6) at admission and was 11.6 mg/L (IQR: 5.9–49.0) at day 10. Among the 73 patients who recovered from COVID-19, the median disease duration between onset of COVID-19 symptoms and clinical healing was 27.5 days (IQR: 17.0–33.2) and the median duration between onset of symptoms and microbiological healing, documented by two consecutive negative nasopharyngeal swabs, was 38 days (IQR: 31.7–50.2).

The duration of SARS-CoV-2 positivity was ≥50 days in a nonnegligible part of patients (17 of 73; 23.3%); such a long interval of viral positivity was described only episodically in China [15,16] yet was reported in a previous case report by our research group [17].

**Figure 1:**
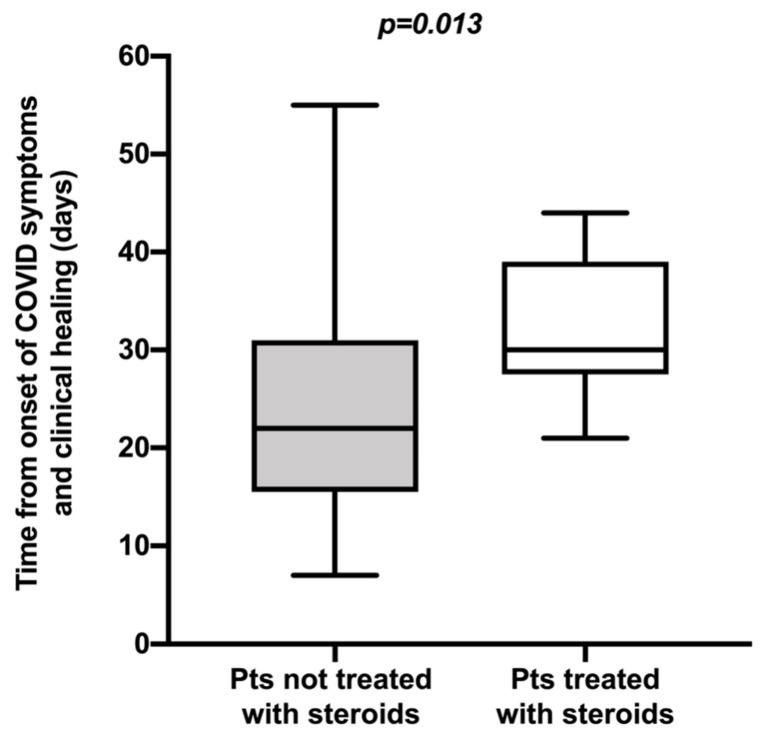
The figure explains the different clinical durations of COVID-19 in patients treated with corticosteroids and not treated with corticosteroids (*p* = 0.013); the bottom of the boxplot indicates the 25th percentile, and the top indicates the 75th percentile. Horizontal lines in the boxes are medians. The lower end of the whiskers represents the extremes of Tukey confidence intervals. The *p*-value reported in the figure is relative to differences between groups and was derived from a linear mixed-effects model. The median value of disease duration in patients treated with use of corticosteroids was 30.0 days (IQR: 27.5–39.0); the median value of disease duration in patients treated without use of corticosteroids was 22.0 days (IQR: 15.5–31.0).

A longer duration of COVID-19 with delayed clinical healing occurred in patients presenting at admission a lower PaO_2_/FiO_2_ ratio (*p* < 0.001), a more severe clinical presentation (*p* = 0.001) and a lower lymphocyte count (*p* = 0.035). Moreover, patients presenting at admission a lower PaO_2_/FiO_2_ ratio and more severe disease showed longer viral shedding (*p* = 0.031 and *p* = 0.032, respectively). In addition, patients treated with corticosteroids had delayed clinical healing (*p* = 0.013) (Figure 1) if compared to patients who had not been treated with corticosteroids. Concerning the patients treated with steroids, they had similar age and comorbidities compared to non-steroid-treated patients. Regarding COVID-19 severity at admission of steroid-treated patients, the median PaO_2_/FiO_2_ was 236.5 (IQR: 142.5–303.7), the median value of lymphocytes was 860.0/uL (IQR: 635.0–1200.0) and C-reactive protein was 87.9 mg/L (IQR: 51.9–137.4). One patient had mild symptoms, 4 patients developed moderate pneumonia and 13 had severe pneumonia. Altogether, these data point out that COVID-19 disease was generally slightly more severe at admission in patients treated with steroids compared to clinical characteristics of the whole cohort of patients (Table 1).

## 4. Discussion

We showed, in a cohort of Italian patients, a long COVID-19 disease duration, predicted by severity of disease, low lymphocyte levels and use of steroids. This study provides several important data from a clinical standpoint. This finding should inform physicians about the clinical phenotype of patients where a long duration of disease and of viral shedding are anticipated and should address pivotal issues in the settings of prevention, duration of isolation and discharge.

However, it is important to underline that detection of viral RNA by RT-PCR does not necessarily equate to ongoing infection. On the one hand, according to the WHO recommendations released on 12 January 2020, a patient could be considered recovered in the presence of two negative RT-PCR results on sequential samples taken at least 24 h apart. In contrast, in a study of patients with mild COVID-19, infectious virus was isolated from nasal-oropharyngeal and sputum specimens during the first eight days of illness but not after this interval despite continued high viral RNA levels at these sites [18].

These findings and other sources [13] of evidence may raise the hypothesis that patients may continue to shed viral RNA in several samples for a long period, but this does not necessarily equate to infectiousness potential. This supports the guidance of 7–14 days self-isolation from symptom onset, but further exploration is required on whether faecal–oral or faecal–respiratory transmission occurs and the role of RNA shedding in virus transmission, especially in severe cases.

While largely indirect data suggest that infected individuals are more likely to be infectious in the earlier stages of infection, other authors have shown that transmission of SARS-CoV-2 occurred mainly after several days of illness and was associated with modest viral loads in the respiratory tract early in the illness, with viral loads peaking approximately 10 days after symptom onset [19].

Therefore, SARS-CoV-2 seems to be transmitted easily, even when symptoms are relatively mild.

As a consequence, despite available data suggesting that prolonged viral RNA shedding after symptom resolution is not clearly associated with prolonged infectiousness [20], it is still unclear how long a person remains infectious.

More recently, on 27 May 2020, the WHO updated the criteria for discharge from isolation as part of the clinical care pathway of a COVID-19 patient, indicating 10 days after symptom onset plus at least 3 additional days without symptoms (including without fever and without respiratory symptoms) for symptomatic patients and 10 days after positive test for SARS-CoV-2 for asymptomatic cases as the timing requested to release a patient from isolation.

The updated criteria for discharge from isolation balances risks and benefits. These recommendations are based on findings of neutralizing antibodies 5–10 days after infection with SARS-CoV-2 and that binding of these neutralizing antibodies to the virus is expected to reduce the risk of virus transmission [21].

Despite these recent recommendations, several countries, including Italy, do not allow discharge and release from isolation only on a temporal basis. While the antibody test is not mandatory, ascertainment of microbiological healing by nasal swabs is still a relevant issue not only for the control of infections in hospitals but also for discharge from hospitals and discharge from isolation management.

The discrepancy in disease duration observed between the data of our cohort and other studies on Asian patients [7,14], within the limits of comparing different populations, may be probably due to genetic and clinical differences of patients and to interindividual differences [22,23], while the hypothesis of relevant mutations in the virus appears unlikely.

In the context of all these considerations and of the residual uncertainty about length of patients’ infectivity, we believe that our findings of a longer clinical and microbiological duration of COVID-19 in our cohort as compared to the existing data is of relevance. Thus, our data should trigger an afterthought in the scientific community to rethink the prevention and treatment models to approaching SARS-CoV-2. In particular, a considerably long COVID-19 duration and SARS-CoV-2 viral shedding and all the relevant clinical and epidemiological consequences should be paid attention, especially considering the need for hospital beds that could be particularly difficult to find in the peak phases of the pandemic, even more so if occupancy of the beds is longer than expected. Moreover, preventive measures and need for isolation could be affected by longer viral shedding and longer disease duration.

About the use of steroids in COVID-19 and its duration, it is important to underline that the association between steroids and severity may be mediated by the baseline status of patients who were treated with these drugs and only a randomized study will clarify a possible cause and effect relationship. As described in the literature, most patients with severe COVID-19 had persistently high levels of erythrocyte sedimentation rate, C-reactive protein, IL-6, TNFα (tumor necrosis factor alpha), IL-1β, IL-8, and IL2R and had associated occurrence of ARDS, hypercoagulation and disseminated intravascular coagulation with clinical manifestations of thrombosis, thrombocytopenia and gangrene of the extremities [24]. Moreover, some authors have proposed a 3-stage classification model, recognizing that COVID-19 disease could show three degrees of increasing severity that correspond to distinct clinical outcomes [25]. We can speculate that, if the viral load is greater, the consequent cytokine storm will be higher, and in these severe patients, the use of corticosteroid drugs may be indicated to try to interrupt this vicious circle, with the possibility, however, of prolonging viral shedding and disease duration, as reported in other settings [26].

A longer duration of viral shedding in patients treated with corticosteroids had also been shown with SARS [27], but only ongoing clinical trials on patients with SARS-CoV-2 will provide the scientific community with solid evidence regarding the use of corticosteroids in COVID-19 and their possible influence on viral shedding and disease duration.

## 5. Conclusions

Our data from an Italian cohort of patients showed that COVID-19 disease duration is predicted by disease severity, lower lymphocyte levels at admission and use of steroids. Disease severity and lower lymphocyte levels at admission also predict longer SARS-CoV-2 viral shedding.

Notwithstanding the many limitations of these study, such as the small sample size, these observations could add new information to the settings of prevention and treatment of COVID-19 patients, contributing to the worldwide battle against the SARS-CoV-2 pandemic.

## Figures and Tables

**Table 1:** Clinical characteristics and parameters of patients recovered from COVID-19.

Clinical Characteristics	Median (IQR)
Age (years)	63 (52.0–76.0)
	Percentage of patients recovered from COVID-19 (*n* = 73) *n* (%)
Gender	
Male	43 (58.9%)
Female	30 (41.1%)
Diabetes	4 (5.5%)
Hypertension	22 (30.1%)
Coronary heart disease	4 (5.5%)
Chronic obstructive pulmonary disease	2 (2.7%)
Atrial fibrillation	7 (9.6%)
Chronic kidney disease	6 (8.2%)
Obesity	7 (9.6%)
COVID-19 severity	
Asymptomatic	1 (1.4%)
Mild symptoms	2 (2.7%)
Moderate pneumonia	33 (45.2%)
Severe pneumonia	34 (46.6%)
ARDS	3 (4.1%)
Treatment with corticosteroids	18 (24.7%)
**Clinical Parameters**	**Median (IQR)**
PaO_2_/FiO_2_ (admission)	242.0 (186.5–304.5)
Lymphocytes (admission)	990.0/μL (682.5–1260.0)
C-reactive protein (admission)	65.0 mg/L (30.8–148.6)
Lymphocytes (day 10)	1320.0/μL (1000.0–1680.0)
C-reactive protein (day 10)	11.6 mg/L (5.9–49.0)
**COVID-19 Duration**	**Median (IQR)**
Days from onset of symptoms to clinical healing	27.5 (17.0–33.2)
Days from onset of symptoms to microbiological healing	38.0 (31.7–50.2)

IQR = interquartile range expressed as Q_1_–Q_3_; ARDS: acute respiratory distress syndrome; PaO_2_/FiO_2_: partial pressure of oxygen in arterial blood/fraction of inspired oxygen ratio.
